# Fractal Dimension of EEG Activity Senses Neuronal Impairment in Acute Stroke

**DOI:** 10.1371/journal.pone.0100199

**Published:** 2014-06-26

**Authors:** Filippo Zappasodi, Elzbieta Olejarczyk, Laura Marzetti, Giovanni Assenza, Vittorio Pizzella, Franca Tecchio

**Affiliations:** 1 Dept. of Neuroscience, Imaging and Clinical Sciences, ‘G. d’Annunzio’ University, Chieti, Italy; 2 Institute for Advanced Biomedical Technologies, ‘G. d’Annunzio’ University, Chieti, Italy; 3 Nałęcz Institute of Biocybernetics and Biomedical Engineering, Polish Academy of Sciences, Warsaw, Poland; 4 Institute of Neurology, Campus Biomedico University of Rome, Rome, Italy; 5 Laboratory of Electrophysiology for Translational neuroScience (LET’S), ISTC, National Research Council (CNR), Fatebenefratelli hospital – Isola Tiberina, Rome, Italy; 6 Dept. of Imaging, IRCCS San Raffale Pisana, Rome, Italy; Medical University of Graz, Austria

## Abstract

The brain is a self-organizing system which displays self-similarities at different spatial and temporal scales. Thus, the complexity of its dynamics, associated to efficient processing and functional advantages, is expected to be captured by a measure of its scale-free (fractal) properties. Under the hypothesis that the fractal dimension (FD) of the electroencephalographic signal (EEG) is optimally sensitive to the neuronal dysfunction secondary to a brain lesion, we tested the FD’s ability in assessing two key processes in acute stroke: the clinical impairment and the recovery prognosis. Resting EEG was collected in 36 patients 4–10 days after a unilateral ischemic stroke in the middle cerebral artery territory and 19 healthy controls. National Health Institute Stroke Scale (NIHss) was collected at T0 and 6 months later. Highuchi FD, its inter-hemispheric asymmetry (FDasy) and spectral band powers were calculated for EEG signals. FD was smaller in patients than in controls (1.447±0.092 vs 1.525±0.105) and its reduction was paired to a worse acute clinical status. FD decrease was associated to alpha increase and beta decrease of oscillatory activity power. Larger FDasy in acute phase was paired to a worse clinical recovery at six months. FD in our patients captured the loss of complexity reflecting the global system dysfunction resulting from the structural damage. This decrease seems to reveal the intimate nature of structure-function unity, where the regional neural multi-scale self-similar activity is impaired by the anatomical lesion. This picture is coherent with neuronal activity complexity decrease paired to a reduced repertoire of functional abilities. FDasy result highlights the functional relevance of the balance between homologous brain structures’ activities in stroke recovery.

## Introduction

The traditional model of control assumes that healthy human physiological systems preserve steadiness by self-regulating their activity reducing fluctuations around a homeostatic equilibrium point. Thus, active controls act to damper fluctuations due to random noise that shift actual operating conditions from the preset states [1]. Differently from this view, a wide bulk of data is now providing evidence that complex fluctuations, observed in several physiological time signals such as heartbeat [2], [3], respiration [4], gait rhythm [2], [5], [6], dynamics of neurotransmitter release [7], electromyography [8], brain activity [9]–[11], are not purely random. Rather they can reveal a temporal organization over multiple time scales. In particular, fractal properties have been described for these signals.

A structure exhibits fractal properties if similar details are observed on different scales [12]. Also a time series can display fractal properties, if statistical similarity emerges at different time scales of its dynamics. A signal is fractal if the scaling properties fit a scale-free behavior, i.e. the same features of small time scales emerge in large ones. This relationship is quantified by the fractal dimension [12].

The power spectrum of these signals, plotted as log- power over log-frequency, follows a descendent straight line (power-law distribution, [13]).

In the light of this evidence, the time-variability properties of the signals, often interpreted as “noise”, could instead have an organized complex structure, reflecting non-linear characteristics of the system that are deeply linked to the underlying organ functioning. Indeed, fractal nature of a signal has often been used to quantify the topological and functional complexity of processes generating that signal [12].

Following this idea, processes underlying brain functions can be characterized by evaluating their complexity using non-linear measures [14]–[18]. Indeed, previous Functional Magnetic Resonance Imaging (fMRI), MagnetoEncephaloGraphic (MEG), ElectroCorticoGraphic (ECoG), and Electroencephalographic (EEG) studies have evidenced direct links between cognitive performance and human brain signal variability (for a review see [19]). In both rest and task conditions, the time variability of the signal has been often interpreted as an index of the system complexity [19], reflecting a wider range of neuronal dynamics, the ability of the system to efficiently adapt to upcoming demand [20], [21], and an efficient itinerancy across phase space of the system (for a review see [19]). As a consequence, the direct measure of the signal complexity can be directly linked to the efficiency of functional abilities [11], [22]. In this frame, the term “complexity” points towards a rich temporal structure recovered in the brain signal, where the system features an intermediate situation between two non physiological limits: pure randomness (e.g. white noise) and strictly periodicity (pure sinusoid) or absolute absence of variability (constancy). Both limits are non-physiological states: the former is related to system dysfunction and the latter is associated to the inability of the system to facilitate state changes, and thus to efficiently process varying and unexpected stimuli.

According to this view, a brain damage can result in a dysfunction of the whole system, that could be reflected in a less complex dynamics of its neuronal activity. As a result, a direct measure of nonlinear dynamics can yield information which improves the ability to distinguish between a physiological and a pathological state. Indeed, the study of nonlinear dynamics of EEG/MEG signals has disclosed new standpoints for the comprehension of normal brain functions and their alterations in several neurological dysfunctions (e.g. Alzheimer disease, schizophrenia, epilepsy [9], [23]–[30]).

After a stroke, the global impairment of the system could originate a transition from a more complex behaviour to a reduced repertoire of repetitive actions. The study of brain activity complexity in the acute phase of stroke is still lacking. In the present work, we aimed to test the sensitivity of fractal dimension analysis of EEG in the acute phase of stroke to two key phenomena: the clinical impairment and the recovery prognosis. We hypothesized a direct link between decreased complexity and a worse general clinical status. Furthermore, we aimed to test the sensitivity of EEG fractal dimension in assessing in acute phase the patient’s ability to recovery. While it is crucial to operate the best of knowledge in limiting the lesion dimension by proper intervention in the first hours after the stroke (http://www.rcplondon.ac.uk/resources/stroke-guidelines), it is a common experience that in the weeks and months following the stroke, a largely inter-individual variable recovery ability occurs despite a nearly identical early clinical picture and similar anatomical size and location of the lesion [31]. In this scenario, individual features with prognostic value in the first week after the stroke onset can help to better understand the pathophysiology of post-stroke recovery. In particular, prognostic measures about recovery ability promise to offer a guide in the path to build personalized rehabilitation treatments, allowing a better allocation of physical therapy and economic efforts. Considering that previous studies revealed a clear neuro-vascular uncoupling in stroke patients [32], [33], neuronal electric activity features per se hold a special usefulness when searching for prognostic markers. Indeed, not only electrophysiological impairments reflect the functional state of neurons surviving cerebral ischemia after mono-hemispheric stroke [34]–[36], but firsts reports of EEG/MEG ability in providing recovery prognosis are also present in literature [37]–[43]. Neuromodulation techniques, able to modify the excitability of specific cortical areas, start to be employed enhancing recovery from stroke [44]–[48]. The ability in changing the intra-cerebral neuronal activity balances by proper non-invasive interventions further strengthen the relevance of electrophysiological prognostic markers, to better indicate the specific alteration to be compensated in individual patients.

Different non-linear measures have been proposed to calculate complexity of EEG signals [49]. However, most of these measures are based on the time-delayed procedures embedding the physiological time series in a multidimensional phase space. In this work, we measured the complexity of EEG time series by their fractal dimension using the method proposed by Higuchi [50] which yields the fractal dimension directly in time domain without necessity of embedding the data in a phase space. Therefore, the algorithm is less time-consuming, overcomes the problems bound to the choice of the embedding dimension, works even with relatively short epochs and is highly noise-resistant. The use of fractal dimension and its robustness in EEG data have been previously demonstrated [51]. Since oscillatory activities in specific frequency bands represent a large part of EEG signal power [52], and EEG/MEG studies demonstrated that oscillatory rest cortical activity changes are highly sensitive to the acute neurological impairment in stroke patients [35]–[36], [53]–[60], in this work the relationship between the oscillatory activity alteration and the fractal dimension of the EEG time series in stroke was also investigated.

## Materials and Methods

### Stroke patients and healthy subjects

Thirty-six patients (mean age 73.0±8.6 years, 12 women) were enrolled in the study after a first-ever mono-hemispheric and mono-lesional ischemic stroke in the middle cerebral artery (MCA) territory. The inclusion criteria were: clinical evidence of sensory-motor deficit of the upper limb and neuroradiological diagnosis of ischemic brain damage in MCA territory. The exclusion criteria were: previous stroke on clinical history; neuroradiological evidence of involvement of both hemispheres or brain haemorrhage; dementia or aphasia severe enough to impair patients’ compliance with the procedures.

Nineteen healthy volunteers, matched for age and gender with patients, were also enrolled as control group (mean age 71.1±6.3 years, 7 females, independent t-test for age between patients and controls: p = 0.512). All subjects were right-handed, as confirmed by the Edinburgh Manuality test, were not receiving any pharmacological treatment at the time of recordings, and resulted normal at both neurological and brain magnetic resonance examinations.

The experimental protocol was approved by the Ethical Committee of ‘San Giovanni Calibita’ Hospital, Isola Tiberina, Rome, and all patients and healthy subjects signed a written informed consent.

### Data collection

Clinical scores, EEG recordings and MRI evaluation were collected in patients between 4 and 10 days (mean 5.4±1.2 days) after the stroke onset (T0). Clinical scores were also collected in post-acute stabilized phase after 6 months (T1). The NIH stroke scale score (NIHss) was used for neurological assessment of stroke severity. The effective recovery (ER) was calculated as ER = (NIHSS at T0−NIHSS at T1)/(NIHSS at T0−NIHSS in healthy).

The EEG activity was recorded by 19 electrodes positioned according to the 10–20 International EEG system (F1, F7, T3, T5, O1, F3, C3, P3, FZ, CZ, PZ, F2, F8, T4, T6, O2, F4, C4, P4) in fronto-central reference, an additional electrode pair served for recording electrooculogram to control for eye blinking. Electrocardiogram was monitored by one bipolar channel placed on the chest. A five minute open-eyes electroencephalographic (EEG) recording was acquired both in patients and healthy subjects at rest, while subjects sat on a comfortable armchair fixating a cross displayed on a screen. Data were sampled at 256 Hz (pre-sampling analogical filter 0.1–70 Hz), and collected for off line processing.

The brain MRI was carried out at 1.5 T in patients and healthy subjects (GyroscanIntera, Philips Medical Systems, Eindhoven, The Netherlands). Spin-Echo, Turbo Spin-Echo, and Fluid Attenuated Inversion Recovery sequences were used. All sequences provided contiguous 5-mm thick slices on sagittal, coronal and axial planes. The identification of the lesion site was performed on axial slices. Lesions were classified as ‘cortical’ (C), if the cortical grey matter was involved and all subcortical structures were spared; as ‘subcortical’ (S), when white matter, internal capsule, thalamus or basal ganglia were affected; and, finally, as ‘cortico-subcortical’ (CS), when both the cortical and subcortical structures were involved. The lesion volume was estimated as follows: in each of the three sets of T1-wheigted sagittal, coronal and axial images, the slice where the maximum size of the lesion appeared was selected, and the maximum diameter (mm) on that image was measured; the total lesion volume was then calculated as the product of these three semi-diameters multiplied by 4/3 π.

### Data analysis

The Reference Electrode Standardization Technique [61], [62] was used to standardize the reference of scalp EEG recordings to a point at infinity that, being far from all possible neural sources, acts like a neutral virtual reference. A semiautomatic procedure based on Independent Component Analysis [63] was then applied to identify and eliminate artefacts (i.e. eye movements, cardiac activity, scalp muscles contraction).

For each EEG channel, the Fractal Dimension (FD) was calculated by means of the algorithm proposed by Higuchi [35]. Fractal dimension was calculated in time windows of 10 seconds and averaged over time. The details of the Higuchi’s algorithm implementation are provided in the Appendix S1. The average of all values obtained for each channel was calculated to obtain a global measure of FD. Fractal Dimension separate values for the right and left hemisphere were also found.

Taking into account the importance of the balance of the neuronal activity between the two hemispheres in recovery from stroke [64]–[67], we estimated a measure of the unbalance between the complexity of the two hemisphere by the inter-hemispheric asymmetry index calculated as follows:
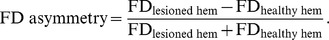



The Power Spectral Density (PSD) was estimated for each EEG channel via the Welch procedure using time windows of 8 s duration (resulting in a frequency resolution of 0.125 Hz), Hanning windowing, 60% overlap, and about 70 artifact free trials. The global PSD was calculated as the mean of the PSDs obtained for the 19 EEG channels. Band powers were obtained in the physiological frequency bands: 0.5–1.5 Hz (sub-delta), 2–4 Hz (delta), 4.5–7.5 Hz (theta), 8–12.5 Hz (alpha), 13–23 Hz (beta1), 23.5–33 Hz; (beta2), 33.5–45 Hz (gamma). The power spectrum (PS) of brain activity roughly follows a straight line when plotted using logarithmic coordinates (log-power vs log frequency):
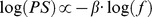



The power low exponent β was estimated as the slope of the fitting line for the PS between 0.5 and 45 Hz by a least-squares linear best-fitting procedure (the mean ± std dev across all subjects of the r-values of the fit was −0.931±0.071). Finally, relative power spectrum (rPS) values were calculated by dividing PS values by the total power between 0.5 and 45 Hz.

Spectral entropy (SE) was estimated according to [68]:
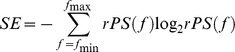
being f*_min_* = 0.5 Hz and f*_max_* = 45 Hz. Spectral Entropy gives a measure of how much a rPS is fragmented in few frequency components (minimal entropy) or flat (maximal entropy), independently of the total power. For example, white noise power spectrum is constant in the whole band, i.e., contains all the frequencies with the same weight, and has maximal entropy; on the contrary a sinusoid, characterized by only one spectral component, has minimal entropy. In summary, spectral entropy quantifies the richness of the spectrum.

### Statistical Analysis

In order to find a topography for the differences of FD values between stroke patients and healthy controls, a repeated measure ANOVA design was performed on FD with *Electrodes* (Fp2, F4, F8, C4, T4, P4, T6, O2, Fz, Cz, Pz, Fp1, F3, F7, C3, T3, P3, T5, O1) as within-subject factor and *Lesion Side* (no lesion, left lesion, right lesion) as between subject factor. Spearman’s correlation was also carried out between FD values and asymmetry index and both NIHss at T0 and NIHss at T1. To check for a possible relationship between FD and clinical recovery, a Pearson correlation between FD and effective recovery was also tested.

Fractal dimension values were then correlated with spectral characteristics of EEG signals. Firstly, differences in global spectral values were investigated in order to confirm the EEG spectral alteration previously observed in stroke patients. To this aim, a repeated measures ANOVA design was applied on mean band power values with *Band* (sub-delta, delta, theta, alpha, beta1, beta2, gamma) as within subject factor and *Group* (Stroke patients, Healthy controls) as between subject factor. Secondly, Pearson’s correlation between FD and band powers, power law exponent and spectral entropy was tested. When needed, Bonferroni correction for multiple comparisons was applied. Finally, a regression analysis with FD values as dependent variable was performed, including all band powers (sub-delta, delta, theta, alpha, beta1, beta2 and gamma) as independent variables.

## Results

NIHss score in patients ranged from 1 to 17 (median: 6.0; 5–95 percentile: 1–15). The ischemic lesion was localized in the left hemisphere in 22 patients and in the right hemisphere in the remaining 14 patients. Right-lesion and left-lesion patients did not differ for NIHss score in acute phase (Mann-Whithney test, p = 0.281), for lesion volume (Mann-Whithney test, p = 0.281) and for age (Independent t-test, p = 0.350). According to the ischemic injury localization, only 1 patient was classified as Cortical, 12 as Subcortical and 23 as Cortical-Subcortical. All patients showed at least some clinical recovery at T1, according to the difference between NIHss at T0 and NIHss at T1 (range 1–12, median: 3.5) and to the ER values (mean 0.67, standard deviation 0.26, range 0.2–1). Ten patients showed a complete recovery (ER = 1).

A strong correlation was found between NIHss at T0 and lesion volume (Spearman’s correlation, rho = 0.610, p = 0.003), while both NIHss at T0 and lesion volume were not correlated with age (Spearman’s rho = 0.223, p = 0.191 and rho = 0.075, p = 0.745, respectively). As expected ER strongly negatively correlated with NIHss in acute phase (Spearman rho = −0.720, p<0.001), and no relationships were found with age, gender and lesion volume.

**Figure 1:**
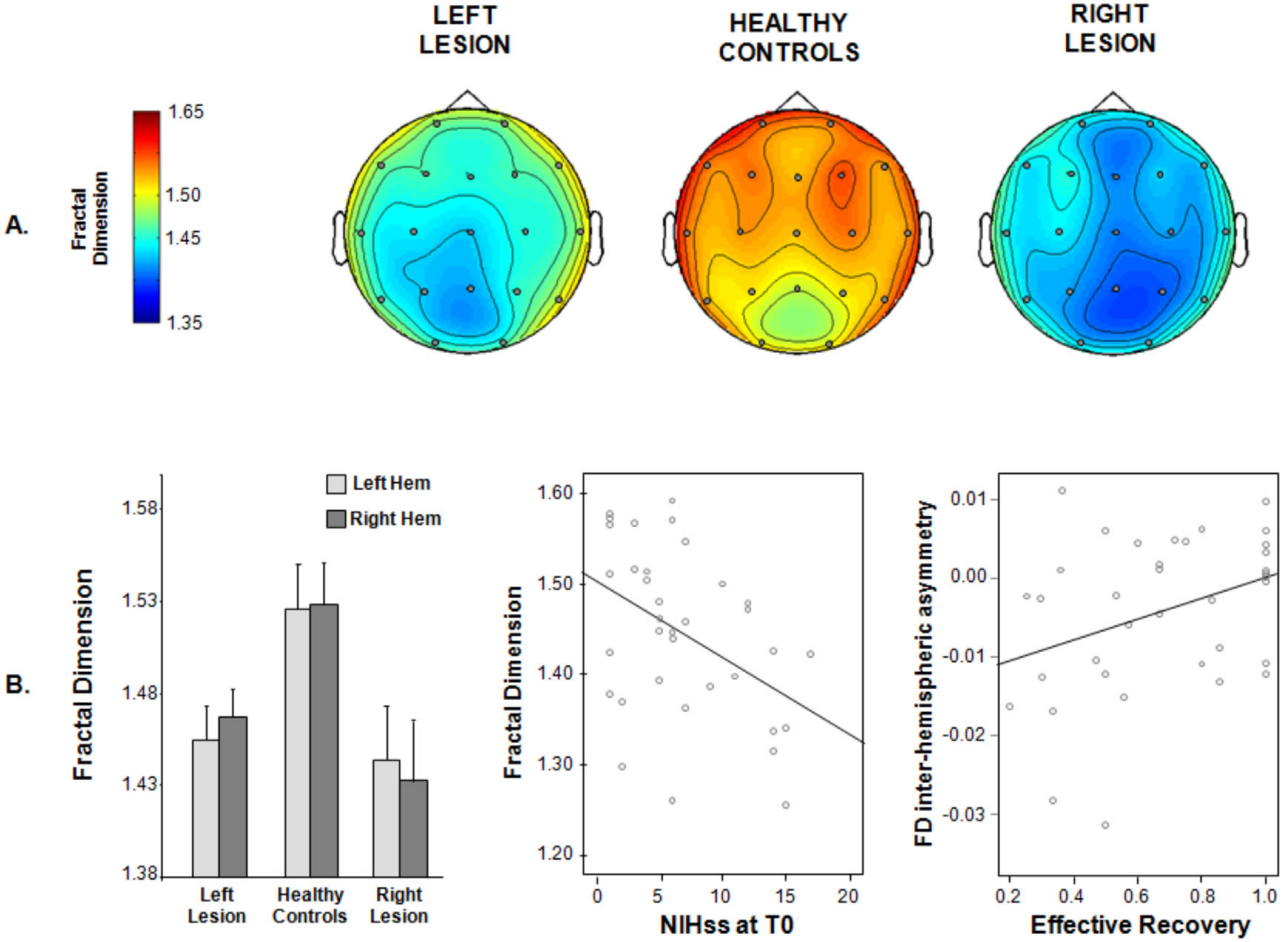
Topography of Fractal Dimension and relationship with clinical status and recovery. A. Topographies of mean values of fractal dimension in the 3 groups: patients with lesion in left hemisphere (left), healthy age-matched controls (center), patients with lesion in right hemisphere (right). EEG electrodes are signed by a full circle. **Left**: mean and standard deviation of fractal dimension, averaged over all sensors of left (Fp1, F3, F7, T3, C3, T5, P3, O1) and right hemisphere (Fp2, F4, F8, T4, C4, T6, P4, O2), in the 3 groups (patients with the lesion in the left hemisphere, patients with the lesion in the right hemisphere, healthy controls). **Center**: scatter-plot of fractal dimension values over NIHss at T0 and fitting line. **Right**: scatter-plot of inter-hemispheric asymmetry index of fractal dimension values over effective recovery and fitting line. To be noted that negative values of asymmetry index denote FD values lower in the lesioned hemisphere than in the non-lesioned one.

The mean value of FD across all EEG electrodes was smaller in patients than in healthy control (1.447±0.092 vs 1.525±0.105, Independent t-test t(53) = 2.829; p = 0.007). Repeated measures ANOVA with *Electrodes* (Fp2, F4, F8, C4, T4, P4, T6, O2, Fz, Cz, Pz, Fp1, F3, F7, C3, T3, P3, T5, O1) as within-subject factor and *Lesion Side* (no lesion, left lesion, right lesion) as between subject factor confirmed this reduction (*Lesion Side* effect F(2,52) = 4.213, p = 0.020) and indicated that the FD difference between patients and healthy controls was not topographically specific (lack of interaction *Electrodes* X *Lesion Side*; p = 0.482). The significance of the factor *Electrodes* (F(18,936) = 6.379; p<0.001) indicated a topography of FD values over the scalp. Indeed, both in patients and in healthy controls FD values were higher in frontal than in parieto-occipital regions (figure 1A).

In patients, a significant difference between the mean values of fractal dimension between hemispheres was found (Paired samples t-test t(35) = −2.422, p = 0.021), with FD values lower in the lesioned than in the non-lesioned hemisphere (mean ± standard deviation 1.446±0.099 vs 1.458±0.094, Figure 1B, left). Fractal Dimension did not correlate with lesion volume (Spearman’s rho = −0.053, p = 0.820) and was not different between patients with (CS) or without (S) cortical involvement (Mann Withney test, p = 0.542).

A negative correlation was found between FD and NIHss at T0 (Spearman’s correlation, rho = −0.426, p = 0.010), indicating a decrease in FD paired to a worse clinical status (Figure 1B, center, Table 1).

**Table 1:** Correlation between FD values and clinical status and recovery.

	NIHss at T0	NIHss at T1	Effective recovery
Global FD	−0.426 (0.010)	n.s.	n.s.
FD lesioned hemisphere	−0.471 (0.004)	−0.367 (p = 0.028)	n.s.
FD healthy hemisphere	−0.381 (0.022)	n.s.	n.s.
FD asymmetry	n.s.	n.s.	0.354 (0.034)

Correlation between the global FD, FD values of lesioned and non lesioned hemisphere and FD inter-hemispheric asymmetry index with clinical status in acute and stabilized phase and clinical recovery. Spearman’s rho (p value) and Pearson’s r (p value) are shown respectively for NIHss and effective recovery. Only values of correlation with p<0.05 are displayed (n.s. = not significant).

The FD asymmetry index measuring the unbalance between the complexity of the two hemispheres was not related to the clinical status at T0, but was related to a worse clinical recovery at T1 (lower values of asymmetry index, i.e. more negative values far from zero, are related to a worse clinical recovery). Indeed, a positive correlation was found between FD asymmetry and effective recovery (Pearson’s r = 0.354, p = 0.034, Table 1, Figure 1B, right).

### Relationship between fractal dimension and spectral characteristics

Differences between patients and healthy controls were found in the mean values of band spectral power over the scalp. Repeated measure ANOVA showed an interaction *Band* X *Group* [F(2.9,151.9) = 5.132; p = 0.002]. Indeed only power in low frequency bands was higher in patients than in controls (Independent t-test: sub-delta: t(53) = −2.728; p = 0.009; delta t(53) = −3.219; p = 0.002; theta t(53) = −3.179; p = 0.002). The increase of power in these bands correlated with a worse clinical status (Spearman correlation between NIHss at T0 and band power: sub-delta: rho = 0.419; p = 0.011; delta: rho = 0.494, p = 0.002; theta, rho = 0.557, p<0.001).

A positive correlation was found between band power in lower frequency bands and FD (Table 2). Indeed, the power-law exponent positively correlated with fractal dimension (r = 0.856, p<0.001). The power-law exponent was also different between patients and healthy controls (−1.47±0.37 vs −1.22±0.32; Independent t-test between the power law of patients and healthy controls:, t(53) = 2.554, p = 0.014) and correlated with a worse clinical status (Spearman’s rho = −0.361; p = 0.031).

**Table 2:** Correlation between Fractal Dimension and Spectral Characteristics.

	All subjects	Healthy subjects	Stroke patients
	*n = 55*	*n = 19*	*n = 36*
Power-law exponent	0.856 (*<0.001*)	0.847 (*<0.001*)	0.896 (*<0.001*)
Spectral Entropy	0.701 (*<0.001*)	0.701 (*<0.001*)	0.704 (*<0.001*)
Sub-delta power	−0.514 (*<0.001*)	−0.667 (*<0.001*)	−0.411 (*0.013*)
Delta power	−0.565 (*<0.001*)	−0.644 (*<0.001*)	−0.510 (*0.001*)
Theta power	−0.618 (*<0.001*)	−0.584 (*<0.001*)	−0.647 (*<0.001*)
Alpha power	−0.729 (*<0.001*)	−0.665 (*<0.001*)	−0.801 (*<0.001*)
Beta1 power	n.s.	n.s.	n.s.
Beta2 power	n.s.	n.s.	n.s.
Gamma power	n.s.	n.s.	n.s.

r-values (p-values) corresponding to the Pearson’s correlation between Fractal Dimension and Spectral Characteristics. Only significant results are displayed.

n.s. = not significant.

Band power values dependence could bias the correlation of spectral values with FD. For this reason, a regression analysis with FD values as dependent variable was performed, including all band powers as independent variables. The result of this analysis showed that only values of power in the alpha, beta1, beta2 and theta entered the model:
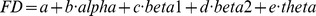
with a = 1.705, b = –0.081, c = 0.058, d = 0.064; e = –0.041. The sign of the coefficients tell us that decrease of FD is associated to an increase of alpha and theta power and to a decrease of power in higher frequencies (beta1 and beta2). The 95% of FD variance was explained by this model, with in particular 53% accounted for by alpha (F(1,53) = 60.156, p<0.001), adjunctive 33% by beta1 (F(2,52) = 155.589, p<0.001), adjunctive 4% by beta2 (F(3,51) = 146.232, p<0.001), adjunctive 5% by theta (F(4,50) = 232.881, p<0.001).

## Discussion

In this work, the complexity of the time-course of EEG signals in stroke patients has been evaluated by means of fractal dimension. One important result of our work is the reduction of fractal dimension in the acute phase of the stroke. This reduction, although found globally over signals from all electrodes, was larger in the compromised hemisphere with respect to the non lesioned one. It has been demonstrated that a stroke lesion, although focal, may disrupt functional communication between the compromised areas and functionally connected remote regions. This communication disruption is linked to functional deficits and overall functional communication has been shown to have a prognostic significance. For example, a loss of interhemispheric functional connectivity at rest in the acute stage correlates with spatial attention and motor impairments and stronger ipsilesional functional connectivity predicts motor improvement [67]. The reduced values of EEG fractal dimension in stroke patients with respect to healthy controls could be the sign that the global loss of complexity, observed for the EEG dynamics of stroke patients and caused by the focal lesion, may indeed reflect the global dysfunction of the system. Nevertheless, the lower fractal dimension of EEG signals from electrodes covering the lesioned areas is a sign of the more severe dysfunction of the peri-lesioned areas. Moreover, the fact that the loss of complexity is bound to a more severe clinical status, is coherent with the idea of a reduced repertoire of functional abilities in patients.

In our patient cohort the unbalance between the complexity of the two hemisphere is related to a worse clinical recovery at six months. The concept of the functional interhemispheric balance in preserving neurological function after acquired brain lesion is supported by results of several studies [64]–[66]. Indeed, a functional inter-hemispheric uncoupling can lead to adverse prognostic consequences [43], [67]. In animal models, a parallel trend between interhemispheric connectivity and neurological severity improvement from acute to chronic stages of cerebral ischemia was recently demonstrated [69] and the prolonged inhibition of contralesional hemisphere within hours following a cortical lesion improves recovery [70].

Ad hoc studies on fractal dimension at the level of cerebral source space are needed to accurately define the spatial specificity of the reduction of the fractal dimension in stroke. Due to the low number of patients, the validity of the clinical value of fractal dimension in stroke is behind the scope of this work, and ad hoc studies are needed to definitely address this point.

In our data, a relationship was also found between fractal dimension and spectral characteristics. Higher spectral entropy values are related to higher fractal dimension values. This suggests that the dynamic of the signals with a spectral richness not depleted by the lesion also shows high levels of complexity in time. This relationship is due to the ability of fractal dimension to capture EEG variation over time at multiple frequencies. To come to the point, the evaluation of the fractal dimension of EEG signals allows to provide a quantitative marker between two extreme conditions: the power of the signal is concentrated in a few frequencies of high amplitude (peaked power spectrum, i.e. low spectral entropy) and the variability of the signal is low (low fractal dimension); the signal power spreads across multiple frequencies (more flat power spectrum, i.e. high spectral entropy), the time course of the signal appears more sharp, and a high fractal dimension is found. The question arises as to whether fractal dimension provides additional information to spectral indexes, particularly in patients where changes in spectral characteristics have been fully described with respect to a healthy sample, and related to clinical status and recovery. Indeed, a mono-lateral ischemic attack induces in its acute phase an asymmetric enlargement in the ipsi-lesional hemisphere of the power oscillation in slow frequency bands (delta and theta [35], [53]–[60]). Increased slow-frequency power within the contra-lesional hemisphere has been also observed in acute phase [38]–[40], [43]. While the slow activity in ipsi-lesional hemisphere is characteristic of perilesional areas and is correlated with a worse clinical status [35], the contra-lesional slow activity in acute phase of stroke is a sign of worse clinical recovery [38], [39], [43]. Although the origin of slow frequency activity in stroke is still debated, evidence supports the hypothesis of a structural and/or functional disconnection at the basis of an enlargement of delta activity both in the peri-lesional areas [71] and in the contra-lesional unaffected hemisphere [43], [72]. On the basis of our result, we could speculate that the alterations in spectral characteristics and in fractal dimension values we found in patients with respect to healthy controls are signs of different kinds of the system dysfunction following the lesion. While slow frequency increase could be a sign of structural and/or functional disconnection, thus responsible for a dysfunction at the system level and for a decreased functional connectivity, the fractal dimension, bound to alpha decrease and beta increase, is a sign of a damage of the regional neural processing at multiscale level.

In our data the high correlation found between lower frequency (delta, theta) and fractal dimension could suggest a direct link between the low frequency increase and the decrease of the fractal dimension. In our opinion, this relationship is only indirect and is mediated by the power-law exponent. In fact, a lower power-law exponent denotes a higher slope of the log-power vs log-frequency plot, i.e. a larger amount of lower frequencies (delta, theta) with respect to the higher (beta and gamma). However, the regression analysis with fractal dimension values as dependent variable and all band powers as independent variables showed that the most of fractal dimension variance (86%) was explained by alpha power increase (53%, negative relationship) and by beta power decrease (33%, positive relationship). These relationships are suggestive of the ability of fractal dimension to summarize the alterations of the neuronal activity in the functional frequency range, in addition to the alteration typical of the disconnection secondary to the brain lesion. The relevance for alpha and beta activities of the fractal dimension is reflected in the topographical distribution of FD values, being lower for parieto-occipital regions, where alpha frequency is most represented, with respect to frontal regions, where beta activity is higher. This topographical distribution has been already described in other EEG studies [51], [73], [74]. In fact, the complexity of the recorded electric signals might be a global marker of the degree of a synergic activity of cortical assemblies of neurons, reflecting in a diffuse synchronization or desynchronization [75].

The fractal dimension reduction was found to be bound to alpha power increase and beta power decrease. Indeed, results of EEG/MEG studies correlated increment of alpha power to inhibition of cortical processing, and inhibition of alpha power to enhanced information processing [76]–[79]. Signs of dysfunctional local hyper-synchronization of neural activity [34], [80], [81] and of a slowing of alpha frequency and enlargement of alpha activity [35], [82]–[85] have been described in stroke patients at the acute phase. On the other hand, in healthy subjects beta power modulation has been observed in sensori-motor processes [86], [87], as well as beta synchronization has been found in several cognitive tasks [88].

Even though the present experiment in its form has not the ambition to provide a new diagnostic tool for clinical stroke diagnostic, our data speak in favor of the use of fractal dimension in clinical practice. Indeed, the application of fractal geometry to neuroimaging allowed to obtain information on scale free properties and modularity, which improve our understanding on brain mechanisms at the systems levels and the dysfunction in neurological diseases [12], [25]. In this light, fractal dimension seems to potentially be a sensitive instrumental marker of stroke disease and recovery capability, and promising to be clinically validated via replication in more numerous and independent patient cohorts.

In conclusion, our small-scale “proof of principle” study documents that the fractal dimension is a good indicator of the complexity of EEG signals, sensitive to clinical impairment in acute phase of the stroke and providing recovery prognosis via its inter-hemispheric asymmetry.

## Supporting Information

Appendix S1 Details of the Higuchi’s algorithm implementation, used to calculate the Fractal Dimension.
(DOC)
