# Supplementary material for: Fractal Dimension of EEG Activity Senses Neuronal Impairment in Acute Stroke
Source: PLoS One. 2014 Jun 26;9(6):e100199. doi: 10.1371/journal.pone.0100199 (PMC4072666; doi:10.1371/journal.pone.0100199)
Supplement: Appendix S1 — Details of the Higuchi’s algorithm implementation, used to calculate the Fractal Dimension. (DOC) [file pone.0100199.s001.doc]

**Appendix**

The algorithm proposed by Higuchi for the calculation of the Fractal Dimension of a time series is based in the direct measure of the mean length of the curve L(k), by using a segment of k samples as measure unit. For different k values, a curve is said to have fractal dimension FD if

$L\left(k\right)\sim k^{FD}$. A.1

FD measures complexity of the curve and so of the time series that this curve represents, ranging from value 1 for deterministic flat curve to value 2 for a stochastic signal, as white noise.

From a given time series of N samples: $y\left(1\right),y\left(2\right),...\text{ }y\left(N\right)$, k new time series are defined:

$y_{k}^{m}\text{: y}\left(m\right),y\left(m+k\right),y\left(m+2k\right),\text{ }...\text{ }y\left(m+\mathrm{int}\left(\frac{N-m}{k}\right)\text{ }k\right)$ A.2

where m is the initial time sample, k the time interval. The length $L_{m}\left(k\right)$ of each curve $y_{k}^{m}$ is calculated as follows:

$L_{m}\left(\text{k}\right)\text{ }=\text{ }\frac{1}{\text{k}}\left[\frac{N-1}{\mathrm{int}\left(\frac{N-m}{k}\right)\text{ }k}\left(\sum_{i=1}^{\mathrm{int}\left(\frac{N-m}{k}\right)} \left|y\left(m+i\text{ k}\right)-y\left(m+\left(i-1\right)\text{ k}\right)\right|\right)\right]$ A.3

$L_{m}\left(k\right)$is not the length in Euclidean sense, but represents the normalized sum of absolute values of differences in ordinates of pair of points of distance k.

For each time interval k, the length of the curve $L\left(k\right)$ is obtained as average over the k sets of $L_{m}\left(k\right)$ values:

$L\left(\text{k}\right)\text{ }=\text{ }\frac{1}{\text{k}}\sum_{m=1}^{k} L_{m}\left(\text{k}\right)$. A.4

The calculation is repeated for k ranging from 1 to $k_{\max}$. If $L\left(k\right)\sim k^{FD}$, then the curve is fractal with dimension FD. In that case, the plot of $\text{log}\left(L\left(k\right)\right)$ against $\text{log}\left(\text{1/k}\right)$ should fall on a straight line with slope equal to FD. Therefore, FD can be obtained by means of a least-squares linear best-fitting procedure:

$F\text{D }=\text{ }\frac{\text{n }\sum\left(\text{x}_{\text{k}}\text{ y}_{\text{k}}\right)-\sum x_{k}\text{ }\sum\text{y}_{\text{k}}}{\text{n }\sum\text{x}_{\text{k}}^{2}-\left(\sum x_{k}\right)^{2}}$ A.5

where $y_{k}=\text{log}\left(\text{L}\left(\text{k}\right)\right)\text{, x}_{\text{k}}=\text{log}\left(\text{1/k}\right),\text{ k}=\text{k}_{1},k_{2},\text{... k}_{\begin{matrix} \text{max} \\ \end{matrix}}$;

n is the number of k values for which the linear regression is calculated, i.e. $2\leq n\leq k_{\max}$.Fractal dimension increases with increase of the value of the k parameter and for k greater than k_max_ it reaches a constant value. Therefore, the point at which FD plateaus is considered as the value of k_max_. In this work k_max_.= 16.

The standard deviation of FD is calculated as:

A.6

where:

$b=\frac{1}{n}\left(\sum y_{k}+FD\text{ }\sum\text{x}_{\text{k}}\right)$ A.7

with standard deviation:

$S_{b}=\sqrt{\frac{1}{n}\text{ S}_{\text{FD}}^{2}\text{ }\sum\text{x}_{\text{k}}^{2}}$. A.8
